# Effects of body mass index and insulin resistance on first-time assisted conception and perinatal outcomes in young polycystic ovary syndrome patients

**DOI:** 10.3389/fendo.2023.1170816

**Published:** 2023-07-24

**Authors:** Jiayu Guo, Yuanhui Chen, Yilin Jiang, Cuilian Zhang

**Affiliations:** ^1^ Reproductive Medical Center, People’s Hospital of Zhengzhou University, Zhengzhou, China; ^2^ Reproductive Medical Center, Henan Provincial People’s Hospital, Zhengzhou, China

**Keywords:** insulin resistance, polycystic ovary syndrome, overweight, early pregnancy loss rate, multivariate logistic regression analysis

## Abstract

**Objective:**

The objective of the study was to explore the effect of body mass index (BMI) and insulin resistance (IR) levels on first-time assisted conception results and perinatal outcomes in young polycystic ovary syndrome (PCOS) patients.

**Design:**

This was a single-center, retrospective, observational cohort study.

**Patients:**

Young women with PCOS undergoing their first embryo transfer were included in the study.

**Main outcome measure:**

Early pregnancy loss rate was the main outcome measure.

**Results:**

The early pregnancy loss rate in the overweight + insulin resistance group (OW+IR group) was significantly higher than that in the non-overweight + non-insulin resistance group (NOW+NIR group) (18.16% *vs*. 9.02%, Bonferroni correction, P = 0.012). The early pregnancy loss rate in the non-overweight + insulin resistance group (NOW+IR group) and overweight + non-insulin resistance group (OW+NIR group) (18.18% and 17.14%, respectively) were also higher than that in the NOW+NIR group (6.07%), but the difference was not statistically significant (Bonferroni correction, all P > 0.05). No significant difference was found in clinical pregnancy rate, live birth rate, and macrosomia rate (all P > 0.05). After adjusting for confounding factors, BMI and IR levels were identified as independent risk factors for early pregnancy loss rate.

**Conclusion:**

BMI and IR levels are independent risk factors for early pregnancy loss in young PCOS patients during the first embryo transfer cycle. Multiple indicators should be considered when assessing pregnancy outcomes, which will promote individualized pregnancy guidance and treatment procedures for PCOS patients.

## 1 Introduction

Polycystic ovary syndrome (PCOS) is a common endocrine disorder in women of reproductive age, accounting for approximately 80% of anovulatory infertility (1). The clinical manifestations of PCOS include sporadic ovulation or anovulation, polycystic ovarian changes, hyperandrogenemia, obesity, and insulin resistance (IR). Many women with PCOS are overweight (OW) or obese (2). Obesity raises the risk of infertility and is a major contributor to the metabolic syndrome. This risk is mostly attributed to the defective hypothalamic-pituitary-ovarian (HPO) axis, low oocyte quality, and decreased endometrial tolerance (3). Obesity is typically accompanied by elevated levels of circulating insulin and a concurrent rise in ovarian androgen production. Extra adipose tissue causes these androgens to aromatize into estrogens, which exerts a negative feedback on the HPO axis and affects gonadotropin (Gn) production. These changes lead to ovulatory dysfunction and menstrual abnormalities. Hyperinsulinemia plays a fundamental role in the pathogenesis of PCOS and is characterized by hypomenorrhea and hyperandrogenemia (4). The symptoms of PCOS are exacerbated by the concurrent presence of obesity, which further raises IR (5). In addition, the increased testosterone (T) production in PCOS causes visceral fat accumulation, which in turn raises IR and hyperinsulinemia, aggravating the vicious cycle (3).

IR is generally considered to be closely associated with obesity. Other studies have found that PCOS patients with IR have a significantly higher incidence of ovulation disorders, anovulation miscarriage, and complications such as gestational diabetes mellitus and gestational hypertension. PCOS patients with IR also exhibit significantly lower conception rates (6–8). However, not much research focused on how IR and BMI levels affect first-time assisted conception results and perinatal outcomes in people with PCOS. Therefore, this study aimed to compare the effects of various levels of IR and OW on first-time assisted conception outcomes and perinatal outcomes in patients with PCOS. Furthermore, the effects of various levels of IR and OW on early pregnancy loss rate were investigated during the first embryo transfer in PCOS patients.

## 2 Materials and methods

### 2.1 Study population

This single-center retrospective cohort study was approved by the Ethics Committee of the People’s Hospital of Zhengzhou University. Retrospective data analysis was performed on PCOS women who underwent their first *in vitro* fertilization (IVF) or intracytoplasmic sperm microinjection (ICSI) procedures at the Reproductive Medicine Center of Henan Provincial People’s Hospital between January 2016 and December 2021. The inclusion criteria were as follows: 1) age ≤35 years; 2) normal ovarian reserve with anti-Mullerian hormone (AMH) >1.1 ng/ml; 3) patients in their first IVF/ICSI-ET assisted conception cycle. The exclusion criteria were as follows: 1) chromosome abnormalities detected on preimplantation genetic screening of preimplantation genetic diagnosis; 2) recurrent spontaneous abortion; 3) uterine cavity abnormalities (uterine adhesions, endometrial polyps, endometritis, unicornuate/bicornuate uterus, etc.); 4) incomplete data cycles, no embryo transfer cycles; 5) thyroid disease, diabetes mellitus, and other systemic endocrine diseases; and 6) uterine fibroids, adenomyosis, and endometriosis. All couples included in the study signed a consent for assisted reproduction treatment (ART) therapy. The study adhered to the fundamental tenets of the Helsinki Declaration.

### 2.2 Diagnostic criteria and formulas

The diagnosis of PCOS was based on the Rotterdam criteria (2003), which requires at least two of the following three criteria: oligomenorrhea and/or anovulation, clinical and/or biochemical signs of hyperandrogenism, and polycystic ovaries on ultrasound scanning (9). IR was assessed using the homeostasis model assessment insulin resistance index (HOMA-IR), HOMA-IR = fasting blood glucose (FPG) (mmol/L)/22.5 × fasting insulin (FINS) (μU/ml). HOMA-IR ≥2.69 was considered IR. According to the criteria of the Chinese Working Group on Obesity, body mass index (BMI) ≥24 kg/m^2^ was considered OW and ≥28 kg/m^2^ was considered obese.

### 2.3 Grouping

PCOS patients were divided into four groups according to HOMA-IR and BMI: Group 1: non-overweight + non-insulin resistance group (NOW+NIR group), Group 2: non-overweight + insulin resistance group (NOW+IR group), Group 3: overweight + non-insulin resistance group (OW+NIR group), and Group 4: overweight + insulin resistance group (OW+IR group) according to IR index and BMI.

### 2.4 Ovulation promotion protocol

The controlled ovulation induction protocol in this study was carried out by the same team based on the patients’ conditions. All of the women underwent either gonadotropin-releasing hormone (GnRH) agonist or flexible GnRH antagonist protocol. After ovulation induction at the appropriate time, oocytes were retrieved by transvaginal ultrasound 36–37 h later according to the fertility center’s protocol, and IVF/ICSI insemination was performed based on the male partner’s condition.

#### 2.4.1 GnRH agonist protocol

For the GnRH agonist protocol, the long-acting GnRH agonist (Diphereline, Ipsen, Tianjin) was injected once at a total of 3.75 mg on the second or third day of the menstrual cycle, 30–35 days after which the serum hormone levels were monitored and the ultrasound was undertaken. On top of that, the same examinations could be accomplished after a short-acting GnRH agonist (Decapeptyl, 0.1 mg/day, Germany Ferring) was injected each day for 14–18 days starting from the middle luteal phase of the previous menstrual cycle. When the requirements for downregulation were met, a dose of 75–300 IU Gn was administered based on age, ovarian reserve, and AMH levels. Depending on the ovarian response and hormone levels, the Gn dosage was modified after 4–5 days. Subcutaneous injection of urinary human chorionic gonadotropin (hCG) was administered when at least two follicles measured ≥18 mm or three follicles measured ≥17 mm. A dose of 4,000–10,000 IU of hCG (Lizhu Pharmaceutical Trading, China) was given to induce ovulation according to the peak estradiol level and age. Extraction of the oocytes was guided by vaginal ultrasonography 36–37 h later.

#### 2.4.2 GnRH antagonist protocol

Ultrasound and basal sex hormone measurements were performed on the second or third day of the menstrual cycle. If no dominant follicles and functional ovarian cysts were observed, Gn was injected to trigger ovulation, starting at the previously mentioned dosage. The size of the follicle and hormone levels were monitored for 4–5 days following Gn treatment. A daily dose of 0.25 mg GnRH antagonist was initiated when dominant follicles showed a mean diameter of 12 mm or estrogen levels ≥200 ng/L or blood luteinizing hormone (LH) levels significantly increased. The process was continued until the hCG dosing day. Subcutaneous injection of urinary hCG was administered when at least two follicles measured ≥18 mm or three follicles measured ≥17 mm. A dose of 4,000–10,000 IU of hCG was given to induce ovulation depending on the peak estradiol level and age. Extraction of the oocytes was guided by vaginal ultrasonography 36–37 h later.

### 2.5 Fresh embryo transfer and luteal support

IVF/ICSI fertilization was performed depending on male semen parameters. After 3–6 days of *in vitro* culture, the best 1 or 2 cleavage embryos or blastocysts were selected according to the routine protocol of our center and then transferred into the uterus under abdominal ultrasound guidance. The embryos that were not transferred were frozen by vitrification with the informed consent of the couple. Fresh cycle transplant patients started receiving luteal support on ovulation day with a progesterone vaginal gel (Crinone, 90 mg/tablet, Merck Serono, Germany) 90 mg/day vaginal medication and dydrogesterone (Duphaston, 10 mg/tablet, Abbott, Netherlands) 10 mg orally bid.

### 2.6 First freeze-thaw embryo transfer and luteal support after whole embryo freezing

The endometrium was prepared by choosing a natural cycle or artificial replacement cycle according to the patient’s condition. In the natural cycle, follicle development and endometrial thickness were monitored by vaginal ultrasound from the 9th or 10th day of the menstrual cycle, and luteal support was given after ovulation (medication as above). One or two cleavage embryos or blastocysts with the best score were transferred at the appropriate time. During the artificial cycle, estradiol valerate tablets (Progynova, 1 mg/tablet, Bayer Pharmaceutical Company, Germany) were taken orally 4–8 mg/day from the second to fourth day of the menstrual cycle, and the endometrial thickness was monitored after 7–8 days. The original dosage was kept the same or increased based on the endometrial thickness. Progesterone was used to transform the endometrium when the medication’s duration was ≥11 days or the endometrial thickness was ≥8 mm. Transforming endometrial medication and embryo transfer strategies are basically consistent with the natural cycle.

### 2.7 Outcomes

Comparison of the general conditions of patients in the four groups: Age, BMI, HOMA, LH, FSH, AMH, years of infertility, and type of infertility

Comparison of assisted conception outcomes in the four groups: duration of Gn, total dosage of Gn, number of oocytes retrieved, number of mature oocytes, number of normal fertilization oocytes, number of available embryos, and blastocyst formation rate

Comparison of perinatal outcomes in the four groups: early miscarriage rate, early pregnancy loss rate, live birth rate, and macrosomia birth rate

The primary outcome of this study was the early pregnancy loss rate. Peripheral blood hCG >50 mIU/ml at 14 days after embryo transfer was used to define pregnancy, and clinical pregnancy was defined as the presence of at least one intrauterine gestational sac 4–5 weeks after transfer (ectopic pregnancy was not included in this study). Biochemical pregnancy was characterized by hCG-positive results in the absence of an intrauterine gestational sac *in utero*. Early miscarriage was defined as a miscarriage occurring within the first 12 weeks of pregnancy. Early pregnancy loss included biochemical pregnancy and early miscarriage. When an early pregnancy loss was determined, all luteal support medications were discontinued. In pregnant patients, luteal support medications were discontinued, and luteal support medications were maintained until 8–10 weeks of gestation. Early pregnancy loss rate = (biochemical pregnancy cycles + early miscarriage cycles)/hCG positive cycles × 100%.

### 2.8 Statistical analysis

All statistical management and analyses were performed using SPSS 24.0 software. The measurement data conforming to normal distribution were expressed as mean ± SD, and one-way ANOVA was used for comparison between groups. All counting data were expressed by percentage (%), and the chi-square test was used to compare the count data between groups. The Bonferroni method was used to compare multiple groups by pairwise comparison. Univariate logistic analysis was conducted to determine the factors affecting clinical outcomes. After adjusting for confounding variables, a logistic regression model was used to investigate the effects of different BMI and IR levels on the outcome indicators. A two-sided P-value <0.05 was considered statistically significant.

## 3 Results

The study initially screened 2,876 patients with PCOS who underwent a first-time embryo transfer cycle, and 1,240 eligible patients were finally enrolled based on the inclusion and exclusion criteria.

### 3.1 Patient demographic and clinical characteristics

**Table 1** shows the demographic and clinical characteristics of the four groups of patients. IR and NIR accounted for 55.65% (690/1,240) and 44.35% (550/1,240) of the total PCOS cases, respectively. OW patients accounted for 54.44% (675/1,240) of all PCOS patients, and 510 cases (75.56%, 510/675) also had IR. NOW patients accounted for 45.56% (565/1,240) of all PCOS patients, and 385 cases (68.14%, 385/565) were NIR. The differences in HOMA-IR (Group 1, Group 2, Group 3, Group 4: 1.73 ± 0.53, 4.00 ± 1.55, 2.02 ± 0.48, 5.11 ± 2.26, respectively, P < 0.001) and BMI (Group 1, Group 2, Group 3, Group 4: 21.06 ± 1.67, 22.29 ± 1.43, 26.54 ± 2.27, 28.27 ± 2.85, respectively, P < 0.001) were statistically significant. AMH, basal follicle-stimulating hormone (FSH), basal LH, and T levels were significantly lower in the OW+IR group compared to those in the NOW+NIR group, whereas the number of years of infertility was significantly higher. No statistically significant differences were observed in age, infertility type, and fertilization method (P > 0.05).

**Table 1:** Comparison of demographic and clinical characteristics of the four groups.

GROUP	Group 1	Group 2	Group 3	Group 4	P
No. of cases	385	180	165	510	
Age (years)	28.91 ± 3.38	28.27 ± 3.55	29.12 ± 3.47	28.51 ± 3.56	0.051
BMI (kg/m^2^)	21.06 ± 1.67	22.29 ± 1.43^a^	26.54 ± 2.27^ab^	28.27 ± 2.85^abc^	<0.001
HOMA-IR	1.73 ± 0.53	4.00 ± 1.55^a^	2.02 ± 0.48^ab^	5.11 ± 2.26^abc^	<0.001
LH (IU/L)	10.85 ± 6.42	9.63 ± 5.47	9.52 ± 5.17	8.51 ± 4.62^a^	<0.001
FSH (IU/L)	6.13 ± 1.44	5.81 ± 1.45	5.90 ± 1.56	5.82 ± 1.55^a^	0.016
T(ng/m)	0.39 ± 0.20	0.43 ± 0.22	0.41 ± 0.18	0.45 ± 0.22^a^	<0.001
AMH (ng/ml)	9.18 ± 4.70	8.87 ± 4.51	8.93 ± 4.92	7.80 ± 4.33^ab^	<0.001
Duration of infertility (years)	3.34 ± 2.07	3.66 ± 2.18	3.56 ± 2.13	4.15 ± 2.41^ac^	<0.001
Type of infertility (%)					0.639
Primary	69.09 (266/385)	66.11 (119/180)	63.64 (105/165)	66.67 (340/510)	
Secondary	30.91 (119/385)	33.89 (61/180)	36.36 (60/165)	33.33 (170/510)	
Methods of ART (%)					0.061
IVF	82.34 (317/385)	84.44 (152/180)	90.30 (149/149)	87.06 (444/510)	
ICSI	17.66 (68/385)	15.56 (28/180)	9.70 (16/149)	12.94 (66/510)	

^1 a,^ Significantly different to Group 1; ^b,^ Significantly different to Group 2; ^c,^ Significantly different to Group 3.

Group 1, NOW-NIR; Group 2, NOW-IR; Group 3, OW-NIR; Group 4, OW-IR.

### 3.2 Ovarian stimulation and first embryo transfer results

As shown in **Table 2**, the starting dosage of Gn, the total dosage of Gn, and the duration of Gn became higher in the OW+IR and OW+NIR groups compared to those in the NOW+NIR and NOW+IR groups, while the number of oocytes retrieved, the number of mature oocytes, the number of normally fertilized oocytes, and the number of normal cleavage embryos decreased. No statistically significant difference was observed in the type of protocol, number of available embryos, number of good quality embryos, and blastocyst formation rate.

**Table 2:** Ovarian stimulation characteristics among the four groups.

GROUP	Group 1	Group 2	Group 3	Group 4	P
No. of cases	385	180	165	510	
Protocol (%)					0.190
GnRH agonist protocol	83.64 (322/385)	85.56 (154/180)	86.67 (143/165)	88.63 (452/510)	
GnRH antagonist protocol	16.36 (63/385)	14.44 (26/180)	13.33 (22/165)	11.37 (58/510)	
No. of basal antral follicles	22.77 ± 4.15	23.18 ± 3.64	22.48 ± 4.61	23.50 ± 3.99^ac^	0.010
Starting dosage of Gn (IU)	121.04 ± 27.62	123.12 ± 25.19	135.45 ± 28.73^ab^	144.44 ± 34.65^abc^	<0.001
Total dosage of Gn (IU)	1631.67 ± 692.83	1696.81 ± 761.10	2245.70 ± 1060.56^ab^	2644.74 ± 1163.04^abc^	<0.001
Duration of Gn (d)	10.98 ± 2.68	10.95 ± 2.74	12.29 ± 3.32^ab^	13.04 ± 3.49^ab^	<0.001
No. of oocytes retrieved	14.87 ± 7.80	14.89 ± 8.61	13.30 ± 7.33	12.85 ± 7.21^ab^	<0.001
No. of mature oocytes	12.68 ± 6.90	12.72 ± 7.43	11.25 ± 6.62	11.08 ± 6.46^a^	<0.001
No. of normal fertilization oocytes	9.08 ± 5.60	9.04 ± 6.01	7.72 ± 4.63^a^	7.83 ± 5.15^a^	<0.001
No. of normal cleavage embryos	8.84 ± 5.49	8.80 ± 5.87	7.50 ± 4.52^a^	7.58 ± 5.08^a^	<0.001
No. of available embryos	7.47 ± 4.97	7.57 ± 5.50	6.89 ± 4.43	6.85 ± 4.77	0.219
No. of good embryos	3.04 ± 3.56	3.46 ± 3.94	2.99 ± 3.23	3.08 ± 3.58	0.633
Blastocyst formation rate(%)	69.98 (1902/2718)	70.99 (893/1258)	68.00 (622/916)	71.98 (2168/3012)	0.087

2 ^a:^ Significantly different to Group 1 ^b:^ Significantly different to Group 2 ^c:^ Significantly different to Group 3.

Furthermore, the early miscarriage rate in the OW+IR group (13.41%) was significantly higher than that in the NOW+NIR group (6.07%) (Bonferroni correction, P = 0.024). The early miscarriage rate in the NOW+IR group and OW+NIR group (13.6% and 12.07%, respectively) was higher than that in the NOW+NIR group (6.07%), but the differences were not statistically significant, and there was no difference in the other pairwise comparisons (Bonferroni correction, all P > 0.05). Similar results were observed in the early pregnancy loss rate: the early pregnancy loss rate in the OW+IR group (18.16%) was higher than that in the NOW+NIR group (9.02%), and the difference was statistically significant (Bonferroni correction, P = 0.012). The early pregnancy loss rate in the NOW+IR group and OW+NIR group (18.18% and 17.14%, respectively) was higher than that in the NOW+NIR group (6.07%), but the difference was not statistically significant (Bonferroni correction, all P > 0.05). There were no significant differences in the other pairwise comparisons. The differences in endometrial thickness, clinical pregnancy rate, and live birth rate were not statistically significant when compared after the first embryo transfer (P > 0.05) (**Table 3**).

**Table 3:** Outcomes of first embryo transfer cycle.

Group	Group 1	Group 2	Group 3	Group 4	P
No. of cases	385	180	165	510	
Type of transfer (%)					0.026
Fresh cycle	43.64 (168/385)	42.78 (77/180)	51.52 (85/165)	52.16 (266/510)	
Frozen cycle	56.36 (217/385)	57.22 (103/180)	48.48 (80/165)	47.84 (244/510)	
No of embryo transferred	1.39 ± 0.49	1.40 ± 0.49	1.49 ± 0.50	1.48 ± 0.50	0.028
Type of transfer embryos (%)					0.008
cleavage	52.73 (203/385)	55.56 (100/180)	65.45 (108/165)^a^	61.96 (316/510)^a^	
blastocyst	47.27 (182/385)	44.44 (80/180)	34.55 (57/165)^a^	38.04 (194/510)^a^	
Endometrium (mm)	10.10 ± 1.93	10.22 ± 2.23	9.87 ± 1.96	10.10 ± 2.12	0.443
Clinical pregnancy rate (%)	64.16 (247/385)	69.44 (125/180)	70.30 (116/165)	64.31 (328/510)	0.190
Early miscarriage rate (%)	6.07 (15/247)	13.60 (17/125)	12.07 (14/116)	13.41 (44/328)^a^	0.028
Late miscarriage rate (%)	4.45 (11/247)	1.60 (2/125)	6.90 (8/116)	8.84 (29/328)^ab^	0.019
Early pregnancy loss rate	9.02 (23/255)	18.18 (24/132)	17.14 (22/124)	18.16 (63/347)^a^	0.010
Live birth rate (%)	55.32 (213/385)	53.33 (96/180)	55.15 (91/165)	47.06 (240/510)	0.060
Single live birth rate (%)	45.45 (175/385)	44.44 (80/180)	43.03 (71/165)	37.65 (192/510)	0.096
Low birth weight rate (%)	6.29 (11/175)	6.25 (5/80)	1.41 (1/71)	6.77 (13/192)	0.400
Macrosomia rate (%)	9.14 (16/175)	11.25 (9/80)	11.27 (8/71)	13.02 (25/192)	0.709
Twin live birth rate (%)	9.87 (38/385)	8.89 (16/180)	12.12 (20/165)	9.41 (48/510)	0.740
Low birth weight rate (%)	52.63 (20/38)	81.25 (13/16)	50.00 (10/20)	56.25 (27/48)	0.208
Macrosomia rate (%)	0	0	0	0	

^2 a,^ Significantly different to Group 1; ^b,^ Significantly different to Group 2.

Birth weight is an important predictor of neonatal and infant survival and a crucial indicator of pregnancy outcome. Low birth weight was defined as fetal birth weight <2,500 g. Macrosomia was defined as birth weight ≥4,000 g. The results of this study indicated that the other three groups may increase the macrosomia rate compared with the NOW+NIR group, but the differences were not significant (**Table 3**).

### 3.3 Univariate logistic regression analysis

The univariate logistic regression analysis revealed that the endometrial thickness on the day of transfer, type of transfer, and groups had statistically significant effects on the early pregnancy loss rate (P < 0.05). In contrast, AMH, age, T, type of infertility, duration of infertility, methods of ART, protocol, type of transfer embryos, and number of embryos transferred showed no statistical significance (P > 0.05). The details are shown in **Table 4**.

**Table 4:** Univariate analysis of effects on early pregnancy loss rate.

	Early pregnancy loss rate		
	B	OR (95% confidence interval)	P
Group1		1	0.012
Group2	0.807	2.242(1.211,4.149)	0.010
Group3	0.777	2.176(1.160,4.082)	0.015
Group4	0.805	2.238(1.346,3.716)	0.002
AGE(year)	0.004	1.004(0.952,1.060)	0.871
AMH(ng/ml)	-0.033	0.968(0.926,1.011)	0.141
T(ng/ml)	-0.184	0.832(0.325,2.131)	0.701
Endometrium(mm)	-0.109	0.897(0.811,0.992)	0.035
Duration of infertility (year)	0.023	1.024(0.945,1.109)	0.568
No. of embryo transferred	-0.252	0.777(0.534,1.133)	0.190
Type of transfer			
Fresh cycle		1	
Frozen cycle	0.471	1.602(1.092,2.351)	0.016
Type of infertility			
Primary		1	
Secondary	-0.013	0.987(0.663,1.470)	0.950
Protocol			
GnRH agonist protocol		1	
GnRH antagonist protocol	0.123	1.130(0.664,1.925)	0.651
Methods of ART			
IVF		1	
ICSI	0.247	1.281(0.731,2.244)	0.387
Type of transfer embryos			
cleavage		1	
blastocyst	0.031	1.031(0.710,1.498)	0.892

### 3.4 Multivariate logistic regression analysis

Multivariate logistic regression analysis was performed to explore the risk factors of early pregnancy loss rate. After adjusting for confounding factors such as age, AMH, T, protocol, methods of ART, endometrial thickness on the day of transfer, type of transfer, and the number of embryos transferred, the results showed that the group was an independent risk factor of early pregnancy loss rate. Compared with group 1, groups 2–4 had significantly higher early pregnancy loss rates (group 2, aOR = 2.377, 95% CI: 1.261, 4.480, P = 0.007; group 3, aOR = 2.304, 95% CI: 1.185, 4.477, P = 0.014; group 4, aOR = 2.229, 95% CI: 1.295, 3.835, P = 0.004). The details are shown in **Table 5**.

**Table 5:** Multivariate logistic regression on the early pregnancy loss rate according to BMI and IR level.

	Early pregnancy loss rate		
	B	aOR (95% confidence interval)	P
Group1		1	0.015
Group2	0.866	2.377(1.261,4.480)	0.007
Group3	0.834	2.304(1.185,4.477)	0.014
Group4	0.801	2.229(1.295,3.835)	0.004
AGE(year)	-0.010	0.990(0.934,1.048)	0.723
AMH(ng/ml)	-0.039	0.962(0.917,1.009)	0.115
T(ng/ml)	-0.582	0.559(0.195,1.599)	0.278
Endometrium(mm)	-0.075	0.928(0.831,1.036)	0.182
No. of embryo transferred	-0.232	0.793(0.528,1.191)	0.263
Type of transfer			
Fresh cycle		1	
Frozen cycle	0.602	1.825(1.166,2.856)	0.008
Protocol			
GnRH agonist protocol		1	
GnRH antagonist protocol	0.109	1.115(0.637,1.953)	0.703
Methods of ART			
IVF		1	
ICSI	-0.180	0.835(0.466,1.495)	0.544

## 4 Discussion

According to epidemiological studies, approximately 50% of PCOS patients are obese, and the prevalence of PCOS is gradually rising among OW or obese people (10). In the present study, IR and OW in PCOS patients accounted for 55.65% and 54.44% of the included cases, respectively, which is similar to the previously reported incidence of IR in Asian PCOS patients (11). Studies have shown that body mass reduction had a corrective effect on reproductive outcomes in obese infertile patients and increased spontaneous fertility in patients with anovulatory PCOS (12). Increased abdominal or visceral fat is associated with IR, which plays a central role in the pathophysiology of PCOS (13), while IR also leads to metabolic abnormalities in women with PCOS (14).

BMI is the most widely used clinical indicator to evaluate OW/obesity. Studies have shown that elevated BMI may lead to alterations in preovulatory follicular fluid metabolites, including elevated levels of insulin, triglycerides, and androgens and decreased hCG levels (15–17). Hassani et al. (18) demonstrated that IR decreases the number of mature oocytes in patients with PCOS by impeding oocyte meiosis and delaying oocyte maturation. In the current study, the duration of Gn and total dosage of Gn were higher in the overweight PCOS group compared with the normal body mass PCOS group, but the number of oocytes retrieved, mature oocytes, normal fertilization oocytes, and normal cleavage embryos were reduced. The OW combined with IR group had the lowest number of these indicators, but the number of available embryos and the number of good embryos were not significantly different.

Earlier investigations indicated that different BMI and IR levels did not impact the outcome of pregnancies. However, the current study confirmed that different BMI and IR levels may affect oocyte maturation, although no discernible effect was found on the number of available embryos, the number of good embryos, the clinical pregnancy rate, and the live birth rate. The differences may be observed in the cumulative pregnancy rate if follow-up statistics are continued.

Similar to previous studies, the present study showed that different BMI and IR levels were associated with early pregnancy loss rates, which were higher in the remaining three groups compared to the NOW+NIR group. The influences persisted after adjusting for factors such as age, AMH, number of embryos transferred, type of transfer embryo, and endometrial thickness. In addition, Li et al. (19) showed that the early spontaneous miscarriage rate was significantly higher and the live birth rate was lower in patients with PCOS with central obesity. Wu et al. (20) showed that weight loss of more than 5 kg may regulate the neuroreproductive endocrine hormone secretion, IR, and gene expression profiles of ovarian granulosa cells. These changes resulted in improved ovarian responsiveness to Gn, the embryo quality, embryo implantation rate, clinical pregnancy rate, and live birth rate and reduced the spontaneous abortion rate in obese infertile PCOS patients undergoing IVF-ET. Chen et al. (8) investigated the effects of IR on the outcome of the first embryo transfer cycle in PCOS patients. The findings of this study revealed that higher IR levels were associated with significantly lower numbers of oocytes retrieved, fewer good embryos, poorer clinical pregnancy rate, and lower live birth rate. In contrast, the rate of early miscarriage and macrosomia increased significantly, and IR was an independent risk factor for early miscarriage and macrosomia in PCOS patients. The hyperinsulinemic state in PCOS patients can stimulate the secretion of androgens from ovaries and adrenal glands, and in the presence of androgen excess, PCOS patients are prone to visceral fat hypertrophy. Moreover, androgens can act directly on oocytes, leading to impaired follicular development and affecting the quality and quantity of follicular cells, ultimately leading to an increased rate of early pregnancy loss in patients with OW+IR. An altered uterine environment may be another factor contributing to the increased early pregnancy loss rate in OW and IR patients (21). Furthermore, because obesity alters Gn pharmacokinetics, obese patients require more Gn to stimulate the ovaries, and higher Gn doses may contribute to their poor pregnancy outcome.

## 5 Conclusion

In conclusion, this study showed heterogeneity in the clinical presentation of basal hormone levels in patients with different levels of BMI and IR, with hyperandrogenemia being more prominent in OW PCOS patients with IR. In terms of first-time assisted conception outcomes, the number of oocytes retrieved, mature oocytes, and normal fertilization oocytes were also significantly lower in patients with OW and IR, while no significant difference was found in the number of available embryos and clinical pregnancy rate, which may be related to the limitations of the retrospective analysis. However, the early pregnancy loss rate was significantly higher in patients with OW combined with IR, and different levels of BMI and IR were independent risk factors for early pregnancy loss rate. Therefore, in clinical practice, multiple indicators should be considered when assessing the pregnancy outcomes of infertile women, in order to promote individualized tailored pregnancy guidance and treatment procedures for young PCOS patients.

## Data availability statement

The original contributions presented in the study are included in the article/supplementary material. Further inquiries can be directed to the corresponding author.

## Ethics statement

The studies involving human participants were reviewed and approved by Reproductive Medicine Ethics Committee of Henan Provincial People’s Hospital. The patients/participants provided their written informed consent to participate in this study. Written informed consent was obtained from the individual(s) for the publication of any potentially identifiable images or data included in this article.

## Author contributions

JG designed and organized the study. YC and YJ were involved in the data extraction and analysis. CZ was responsible for providing data and guiding research. All authors listed have made a substantial, direct, and intellectual contribution to the work and approved it for publication.
